# Learning from eavesdropping on human-human encounters changes feeding location choice in horses (*Equus Caballus*)

**DOI:** 10.1007/s10071-025-01946-1

**Published:** 2025-03-17

**Authors:** Konstanze Krueger, Anika Roll, Anna J. Beyer, Angela Föll, Maren Bernau, Kate Farmer

**Affiliations:** 1 Department of Equine Economics, Faculty of Agriculture, Economics and Management, Nuertingen-Geislingen University, Neckarsteige 6-10, 72622 Nürtingen, Germany; 2 Zoology/Evolutionary Biology, University of Regensburg, Universitätsstraße 31, 93053 Regensburg, Germany; 3 Centre for Social Learning & Cognitive Evolution, School of Psychology, University of St Andrews, St Andrews, Scotland KY16 9JPh UK

**Keywords:** Horse, Human impact, Individual learning, Social eavesdropping, Social ecology, Social experience, Social learning

## Abstract

When animals observe human signals, they may learn from them. Such learning from humans has been reported for intentional communication between humans with animals, but animals might also learn socially by observing unintentional information transfer when eavesdropping on humans-human encounters. In this study, 12 of 17 horses significantly changed their preference for a feeding location after observing approval in a human-human interaction there, and horses kept in social housing adapted in a higher percentage of trials to human-human demonstrations than those in individual housing. This indicates, for the first time, that some animals change their feeding strategies after eavesdropping on human-human demonstrations and that this adaptation may be dependent on social experience. As horses maintained the observed preference for a feeding location when the demonstrators were absent, we suggest that they learned by applying individual and social learning mechanisms. The horses social rank, age and sex did not affect their learning performance. However, particular demonstrators tended to have a stronger impact on the horses’ performance. Future research should further investigate the durability of this preference change in the absence of repeated demonstrations, and establish whether long-term social learning sets in. This would have important implications for unintentional long-term impacts of human interactions on interspecies communication.

**Supplementary Information:**

The online version contains supplementary material available at 10.1007/s10071-025-01946-1.

## Introduction

When animals observe humans, they may learn about context specific signals and values through social information transmitted by the persons’ positions and actions (Zajonc 1965; Heyes 1994; Whiten et al. 2004; Laland 2004; Laland and O’Brien 2011; Sueur and Huffman 2024). Therefore, we examined the possibility of animals learning socially about feeding location values through eavesdropping, e.g. through the unintentional transfer of information when animals observe a human-human interaction (Kundey et al. 2010; Freidin et al. 2013; Ridley et al. 2013). We investigated whether animals adapt their feeding location choices after observing human-human feeding encounters, even in the absence of the humans involved. Such social learning of human eating strategies has been discussed for Japanese macaques, which demonstrate the washing of potatoes before eating them (Nakamichi et al. 1998; Fiore et al. 2020).

Social learning of feeding strategies has previously been reported between humans and a number of species where information has been communicated directly and intentionally (Spottiswoode and Wood 2023; Sueur and Huffman 2024). For example, human bee-hunters in central Africa are assisted by honey-guide birds that lead them from tree to tree in search of beehives. The people then open the beehives, collect the honey, and leave the wax and the larvae to the birds. To team up with the hunters, the birds learn the calls specific to the people of the regional cultures (Spottiswoode and Wood 2023).

While this example shows intentional transfer of feeding information, information may also be transferred unintentionally through eavesdropping, by which an uninvolved third party (Schneider and Kruger 2012) adapts its behaviour on the basis of information transmitted between two interacting parties (Kundey et al. 2010; Freidin et al. 2013). For example, after eavesdropping on a human-human eating encounter, dogs preferentially approached the person who they had observed being treated positively with the words and gestures of another person (Freidin et al. 2013), and a person they had observed behaving positively towards another person (Kundey et al. 2010). However, in both these cases the dogs’ preferences were shown in the presence of, and towards, the observed human interaction partners (Kundey et al. 2010; Freidin et al. 2013). It remains unclear whether social learning may subsequently set in to produce long lasting, stable feeding strategies (Byrne and Whiten 1988; Byrne 1994; Heyes 1994; Whiten et al. 2004), as demonstrated by potato washing Japanese macaques (Nakamichi et al. 1998; Fiore et al. 2020).

Social learning capacities have been proposed to be well developed in some species, especially in animals living in highly complex social environments (Byrne and Whiten 1988). Horses are a good model organism for studying whether, and under which conditions, animals learn from unintentional information transfer through human-human encounters. Horses live in complex social environments in natural environments and when kept in social groups, but in limited social environments when kept in individual housing, which may compromise their welfare (Krueger et al. 2021) and cognitive capacities, such as responding to human given cues (Liehrmann et al. 2023). Group-living horses learned socially to operate a feeding device (Krueger et al. 2014; Bernauer et al. 2020) after direct demonstrations by familiar conspecifics (Krueger et al. 2014) and humans (Schuetz et al. 2017; Bernauer et al. 2020).

Furthermore, horses have been shown to use human given signals and learn from individual persons in several situations. For example, Clever Hans, the horse which was erroneously considered to have the mathematical skills of a 12-year-old child, responded to the unintentional, human expressions and gestures, which signaled whether Hans should start or stop tapping his hoof. Hans used the signals of his owner, and, when the owner was not present, of persons in the audience who knew the answer (Pfungst 1907). Horses orientate on the direction of human attention (Krueger et al. 2011), and use human pointing gestures (Miklósi and Soproni 2006; Maros et al. 2008; Liehrmann et al. 2023), voice commands (Proops and McComb 2010) and body orientation (Proops and McComb 2010; Proops et al. 2013) to find food. They have also learned to use symbols to communicate with humans when they wanted rugs be taken off or put on (Mejdell et al. 2016).

To reduce the costs of social learning, horses form adaptation and learning strategies (Laland 2004). They adapted to the attention of familiar rather than unknown persons (Krueger et al. 2011) and preferred to feed from buckets from which dominant horses had fed before (Krueger and Flauger 2008). Younger, lower-ranking horses learned socially from older higher-ranking horses (Krueger and Heinze 2008; Krueger et al. 2014).

Here we examined whether eavesdropping on human-human encounters that expressed approval and disapproval for particular feeding locations may cause domestic horses to change their preference for feeding locations and whether the learned preference would persist in the absence of the demonstrating persons. If so, this would show for the first time that human to human interactions may have an unintentional, long-term impact on animals’ behaviour. We asked: (a) Do horses change their preferred feeding locations after eavesdropping on disapproval and approval in a human-human interaction? (b) Does the learned preference persist in the absence of those people? (c) Do the housing conditions or the social rank, age, or sex of the horses affect their feeding location choice?

## Materials and methods

### Research area

The study was conducted in five private horse management facilities in the German states of Bavaria and Baden-Württemberg between 10th May, 2021 and 29th September, 2022. Riding arenas or round pens were used as the experimental areas and, in each case, these were close to the stables.

### Animals

Seventeen horses took part in the experiment, eight females and nine castrated males, with a median age of 12 years old (min. 4 years, max. 28 years). They were of mixed breeds: three thoroughbreds, four warmbloods and ten ponies (Table 1). All horses were healthy, with a medium body condition score of 3 on a scale of 1 (emaciated) to 5 (obese). Their welfare was monitored in annual routine health checks by the animal welfare commissioner of the research organization.

Fourteen of the horses lived in social housing, in open stabling. Three of the test horses were kept in individual housing, two in paddock boxes and one in a single box with turnout (Table 1). The length of turnout varied between the facilities, but all were turned out for at least one hour per day in winter, and three hours per day in summer. The bedding in the boxes consisted of straw and the boxes were cleaned daily. The feed of the test animals comprised roughage ad libitum, salt, and individual amounts of surplus feed. Water was accessible ad libitum. In addition to free movement, the horses also had at least one hour of controlled exercise, such as walking, lunging, or riding, every day.

### Experimenters

**Fig. 1:**
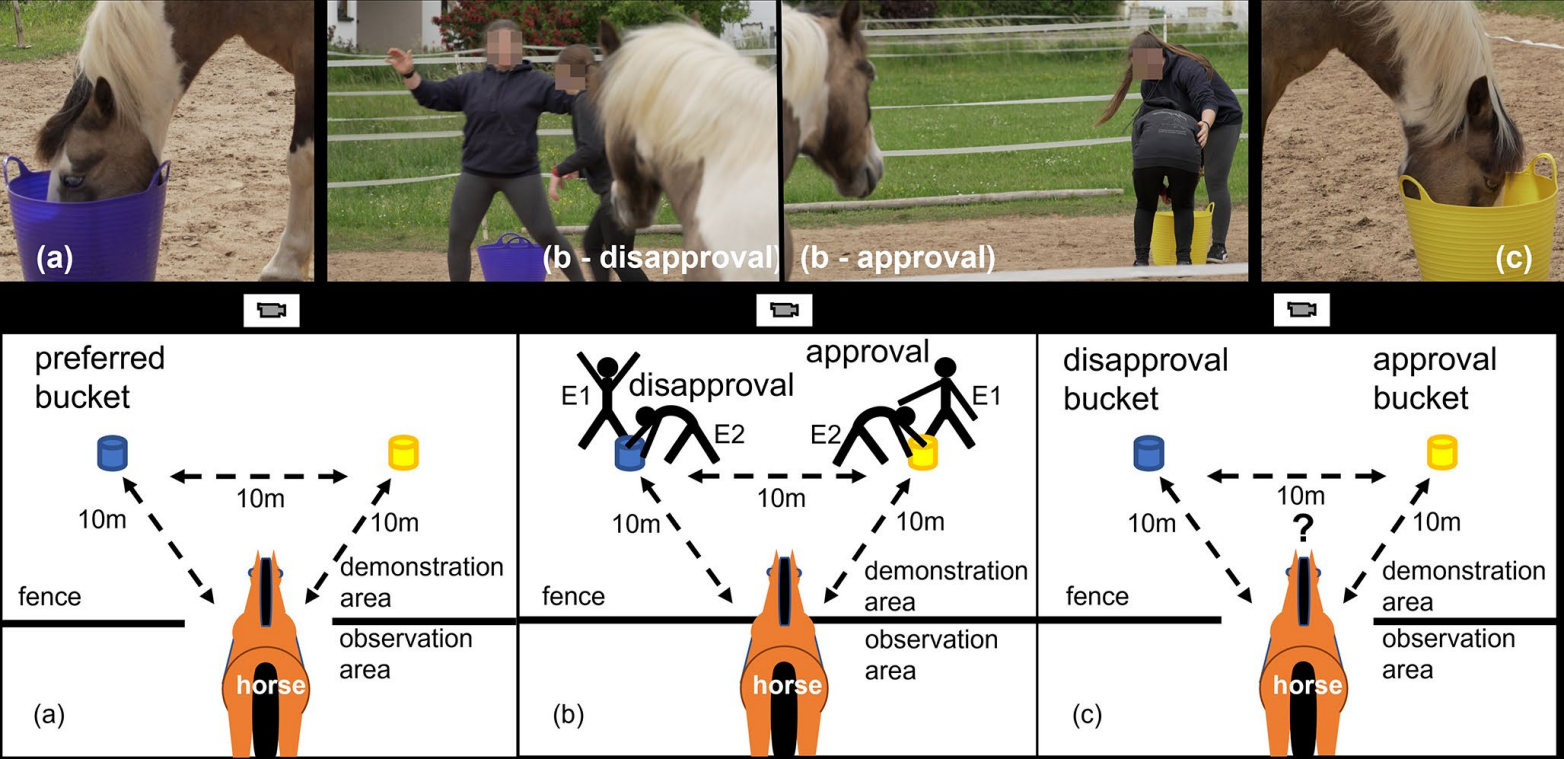
Experimental procedure of the bucket preference test. As horses are a dichromatic species (Carroll et al. 2001), blue and yellow buckets were used to make a clear distinction between the two locations. The buckets were in the same, equidistant positions (10 m), throughout the experiment for each test horse. A camera for documentation was placed outside of the experimental areas. (**a**) Pre-observation bucket preference test: Horses were allowed to choose which bucket to approach and feed from in the demonstration area. The preferred feeding bucket and location, was designated the disapproval bucket for the demonstration phase, e.g. the blue bucket in the left position (photo a). (**b**) Demonstration and observation phase: Horses observed from the observation area the disapproval (photo b – disapproval) or approval (photo b - approval) of a familiar person (E1) towards another person (E2) who took and ate a piece of food from each bucket in the demonstration area in random order. Note: the photo b – disapproval was taken after the E2 had taken a piece of food from the blue bucket. (**c**) Post-observation bucket preference test: after the demonstrations, the fence between the observation and demonstration area was opened and the observing horse was free to choose to eat from either the disapproval bucket or the approval bucket, e.g. the yellow bucket at the right position (photo c)

Two male and nine female persons, eleven in total, participated in the experiments in three experimental roles. Three persons participated in stable A and four in stable B. Three persons participated in the stables C-E and, when switching stables, these experimenters changed roles (Fig. 1b).

#### Experimenter 1 (E1)

was well known to the respective horse and was the principal caretaker, who had fed, looked after, and worked with the horse on a daily basis for at least one year. We chose a familiar person for this role because horses were shown to adapt their attention (Krueger et al. 2011) and their behaviour (Krueger and Heinze 2008), and to learn to operate feeding apparatuses from familiar persons (Bernauer et al. 2020). Five female persons participated in this role, one at each stable. E1 displayed either disapproval or an approval (Fig. 1b) when demonstrator 2 (E2) ate a piece of carrot from one of two feed buckets. E1 also recorded the social behaviour of horses in the field before the experiments started in the facilities offering social housing.

#### Experimenter 2 (E2)

was either little known or completely unfamiliar to the test horse. Four female persons participated in this role, with one performing in two stables. E2 approached one of two feed buckets and ate a piece of carrot from it (Fig. 1b), and then received either approval or disapproval from E1.

#### Experimenter 3 (E3)

was either little known or unknown to the horse. Two male and seven female persons participated in this role. He or she helped with the experimental setup, and with the experimental procedure by operating the camera, bringing the test horse into the observation area, opening the gates of the observation area, and returning the horse to the observation area for upcoming trials, or to the stables when the session was terminated, He/she refilled feed buckets as necessary and recorded test results on paper.

### Experimental area

The experimental area was in the riding arena or round-pen in each of the facilities. The arena was well known to all the test horses and was close to the stabling. The test horses were able to stay in visual contact with their stable mates, but the stable mates could not see the experimental procedure. The experimental area was set up so that the horses were led into the observation area, and could only enter the test area from the observation area.

An experimental area of 20 m x 20 m was divided into an observation area (5 m x 20 m) and a demonstration area (15 m x 20 m) with a temporary horse fence (Fig. 1). A gate between the observation and the demonstration area was made in the centre of the fence, so that the horses could enter the demonstration area without side bias. A camera was placed outside the test area, facing the gate and set to record the whole test area.

Two buckets were placed in the demonstration area forming a triangle with the entrance, with 10 m between each point (Fig. 1). One bucket was yellow and the other one blue to ensure the horses could clearly distinguish between the two buckets, as horses are dichromats and can distinguish the peak wavelengths of the colours blue and yellow (Carroll et al. 2001). The buckets were kept in permanent positions because the colour was intended to be combined with local enhancement effects for specific feeding areas, as applied in dogs (Jim et al. 2020) and horses (Devenport et al. 2005; Krueger and Flauger 2008; Hothersall et al. 2010).

### Experimental procedure

#### Habituation phase

On the day before the pre-observation preference test, E3 led each horse into the observation and then into the demonstration area, led it to the feed buckets containing pieces of carrot and a small quantity of horse feed, and allowed it to eat from the buckets. When horses approached and ate from both buckets without hesitation, E3 brought them back to the observation area and the gate to the demonstration area was closed. Then, the lead rope was removed and the gate was reopened to allow the horse to enter the demonstration area alone and feed from the buckets. This whole procedure was repeated for a median of 10 trials (min. = 3 trials, max: = 16 trials), until the horse walked spontaneously towards the buckets.

#### Pre-observation bucket preference test

(Fig.  1a). As in the habituation phase, the horse was led in the observation area, the lead rope was removed, then the gate was opened to allow the horse to enter the demonstration area alone and feed from its choice of bucket. Both buckets always contained pieces of carrot and a little supplementary feed. The procedure was repeated for 18 trials over three days (six trials each day), and the horses’ feeding location choices were recorded by E3 on video and on paper.

#### Demonstration and observation phase

(Fig.  1b). For the observation phase the horse’s preferred bucket and location in the pre-observation test became the disapproval bucket, and the other location was assigned as the approval bucket. If a horse displayed equal bucket preference in the pre-observation preference test, the disapproval and approval buckets were assigned at random.

E3 brought the horse into the observation area, from which it could observe a human-human interaction at each of the buckets. The experimental set up was similar to the habituation and feed bucket preference test, but now, E1 stood at a randomly selected feed bucket. E2 then entered the demonstration area through the gate, walked to the feed bucket where E1 was standing, took a piece of carrot from the bucket and ate it.

If E1 was standing at the disapproval bucket, she showed disapproval to E2 in the manner she would typically show disapproval to a horse, and if E1 was at the approval bucket, she showed approval to E2 as she would show approval to a horse. Therefore, the words, facial expression and gestures slightly differed between the particular E1s’. In general, the demonstration was as follows:

- Disapproval: E1 stood upright, looking and speaking directly at E2, waved her arms, and said in a harsh manner “no-no, don’t do that” (or individual variations) until E2 retreated.
- Approval: E1 would bow towards the eating E2, touch her shoulder, and say in a gentle manner “good, nice job” (or individual variation), while E2 continued eating.

The demonstration was repeated six times in succession on each day. After the sixth demonstration, E1 and E2 left the demonstration area.

#### Post-observation bucket preference test

(Fig.  1c). After E1 and E2 had left, and the buckets were replenished, E3 opened the gate to allow the horse to enter and approach a bucket. Its choice was documented by E3, who then returned the horse to the observation area. Each horse was then given up to nine further trials with no additional demonstrations. If the horse lost interest and refused to cooperate, the trials were suspended for that day and the whole procedure, including the demonstrations, was repeated the next day. If the horse did not show interest in the test on the following day, the test was finalized for this particular horse.

The demonstration and observation, and post-observation procedures were repeated for a maximum of 8 days. If a horse showed an 80% or higher bucket preference for at least two consecutive days, the horse was considered to have reached the performance criterion. For two horses the test was terminated before the 8th test day out of loss of interest, even though the horse reached the performance criterion only once.

### Social behaviour

One week before the commencement of the experiments at each of the facilities, E1 observed the social behaviour of the horses that were in social housing. 15 h of continuous observations per group were distributed evenly over the daylight hours of at least 3 days within one week. The frequency of agonistic behaviours displayed between the horses was collected by continuous recording ad libitum (Martin and Bateson 2021) and recorded on paper. Agonistic behaviours were defined as threats to bite or to kick, bites, kicks, chases, retreats, and approaches which elicited a retreat of the approached horse (McDonnell and Haviland 1995; McDonnell 2003). Social ranks were then calculated from these data, as detailed under Data evaluation – social rank below.

### Data

#### Data recording

The horses’ behaviour during the pre- and post-observation trials was recorded by five different persons (the five E1s) at the testing locations, both on paper and with either a Canon EOS 60D camera or a GoPro Hero 3 camera. After the experiments, E1 compared the video documentation with the paper recordings, transferred the data on an excel sheet. In addition, for an inter-observer reliability assessment, an independent, naïve person transferred the data to a second raw data sheet. The naïve person’s rating of the horses’ choices to feed from either the approval or the disapproval bucket correlated 100% with the rating of the three E1s (Kendall’s rank correlation tau: *N* = 794, *p* < 0.001).

#### Data evaluation - performance

Regression analysis was used to assess whether horses learned to choose a particular bucket as follows: The individual horses’ performances when choosing feed buckets in the pre-observation phase and the daily post-observation trials were analysed to assess the development of a preference for a particular bucket. Only horses that showed a significant increase in choices for the approval bucket (Regression analysis: *p* < 0.05, Supplementary Material) were considered to have learned to choose the approval bucket.

#### Data evaluation - social rank

The horses’ social ranks were analysed as has been previously described (Krueger et al. 2012), and are shown in Table 1. We calculated the social rank of each animal from their agonistic encounters with the help of the average dominance index (ADI) as follows. The dominance index per pair of individuals, wij, is the frequency individual i won against opponent j divided by the total agonistic encounters between the pair, thus wij = xij / (xij + xji). Wins were counted for the initiator of an encounter when an approached or challenged animal retreated for one step or more. We excluded a pair from the analysis if the two individuals were not involved in an encounter. The average dominance index of an individual is the average of all its dominance indices with all its interaction partners, thus 1/N Σ j wij. The ADI can range between 0 and 1. Therefore, a higher value indicates a higher rank in the hierarchy (Hemelrijk et al. 2005).

Not only the type of agonistic behaviour, but also the reaction of the receiver is decisive in counting wins and losses. For example, an animal may respond by retreating both when it is being kicked and when it is approached. In both cases the receiver loses and the initiator wins. This method enables all agonistic behaviour types to be used, irrespective of their frequency, and provides the largest possible sample size for the rank evaluation [28]. We chose the ADI for its reliability and computational simplicity. Simulations showed that the ADI can deal with missing data between pairs of animals and still provides more reliable results then comparable dominance assessment methods (Hemelrijk et al. 2005).

#### Data analysis

Statistical analysis and the depiction of the data (Table 1; Supplementary Information Tables S1) was conducted using R Studio (version 0.99.484, Boston MA, USA) of the R-Project statistical environment (R Development Core Team, version 4.4.2) and the package lme4. Most of the data were not normally distributed (K-S test). A Kendall’s rank correlation tau test was applied for an inter-observer reliability assessment between the experimenters and a naïve person. Regression analyses were used to evaluate the horses’ learning performances at individual level. A Friedman rank sum test and Wilcoxon signed rank tests were used to compare the horses’ learning performances at group level. We applied a nested Generalised Estimation Equation (GEE) for multivariate factor analysis, nested for the individual horses as a random effect. All other factors under consideration were not truly independent from the experimental design and were therefore included as fixed factors. The GEE to analyse the likelihood of factors affecting the choice for a certain feed bucket throughout the total trials (*N* = 794) was applied, with the following formular: GEE (formula = choice.disapproval.vs.approval bucket ~ (ADI.horse + age.horse + day + handling.person.E1 + housing + sex.horse + strength.bucket.preference.habituation + trial) %in% horse.num, family = binomial(logit), data = Dataset). The model with the best fit (the model with the lowest information loss versus the lowest clustering, quoted with the lowest Akaike Information Criterion, AIC) was chosen after stepwise removal of factors with the following formula: GEE (formula = choice.disapproved.vs.approved ~ (day + handling.person.E1 + housing + ADI.horse) %in% horse.num, family = binomial(logit), data = Dataset). For the full and reduced model see Supplementary Information File S1. All tests were two sided, and the significance level was set at *p* ≤ 0.05. After multiple testing, the significance level was adjusted with a Sequential Bonferroni Correction after Holm (1979) and only p-values that were below the corrected significance level were considered to be significant.

## Results

### Feed bucket preference

**Fig. 2:**
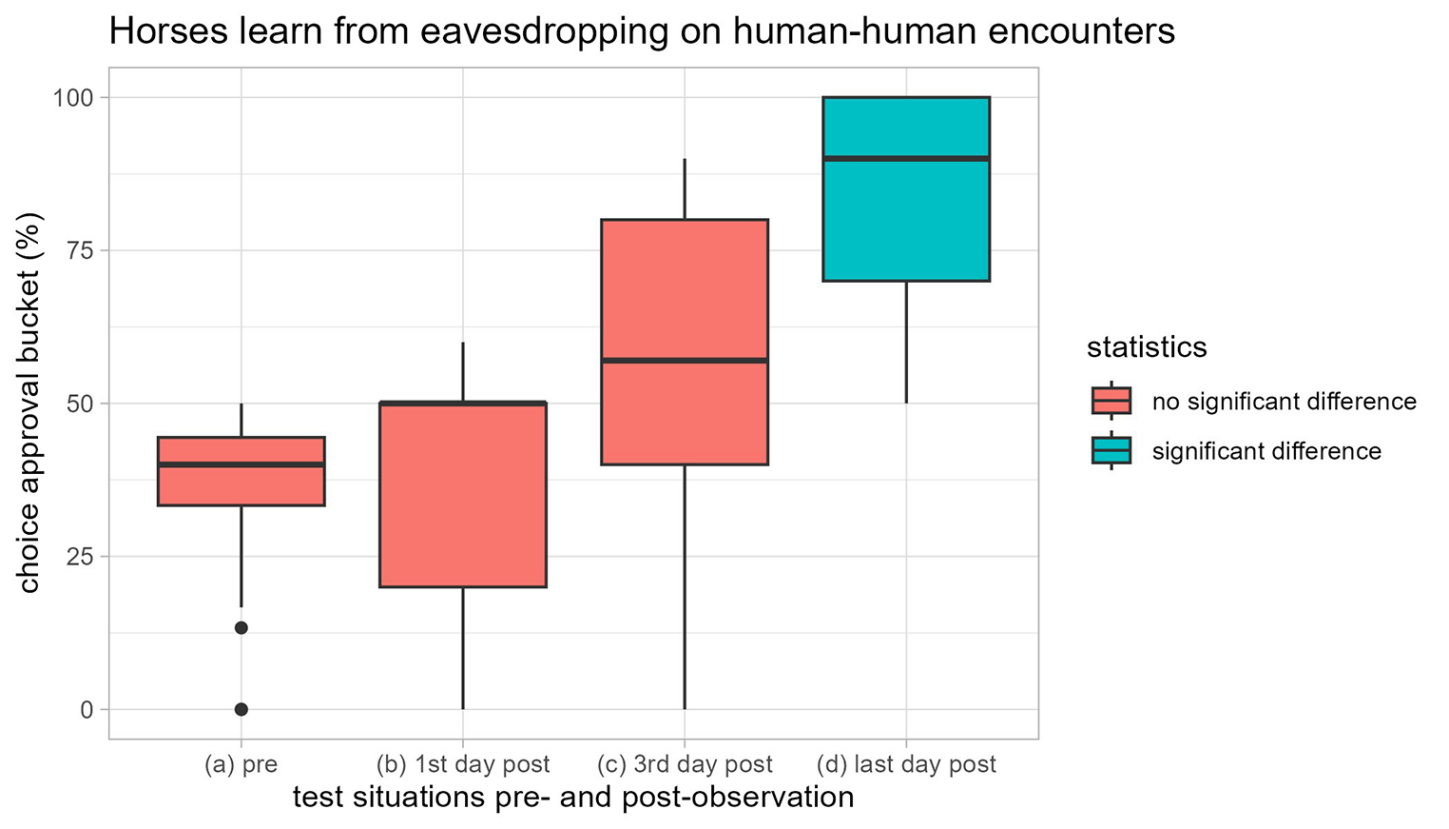
The horses’ group performance in learning to feed from the approval bucket after observing human-human encounters. The group of test horses shows a significant preference for the approval bucket on the last test day post observation (**d**), which differs from all previous group performances (blue; Wilcoxon signed rank tests: *N* = 17, all p < than sequential Bonferroni corrected significance level). However, the preference of the group of test horses for the approval bucket does not increase insignificantly, and there is large individual variation from (**a**) pre-observation, to (**b**) the first day, and (**c**) third day (red; Wilcoxon signed rank tests: *N* = 17, all p > than sequential Bonferroni corrected significance level)

**Fig. 3:**
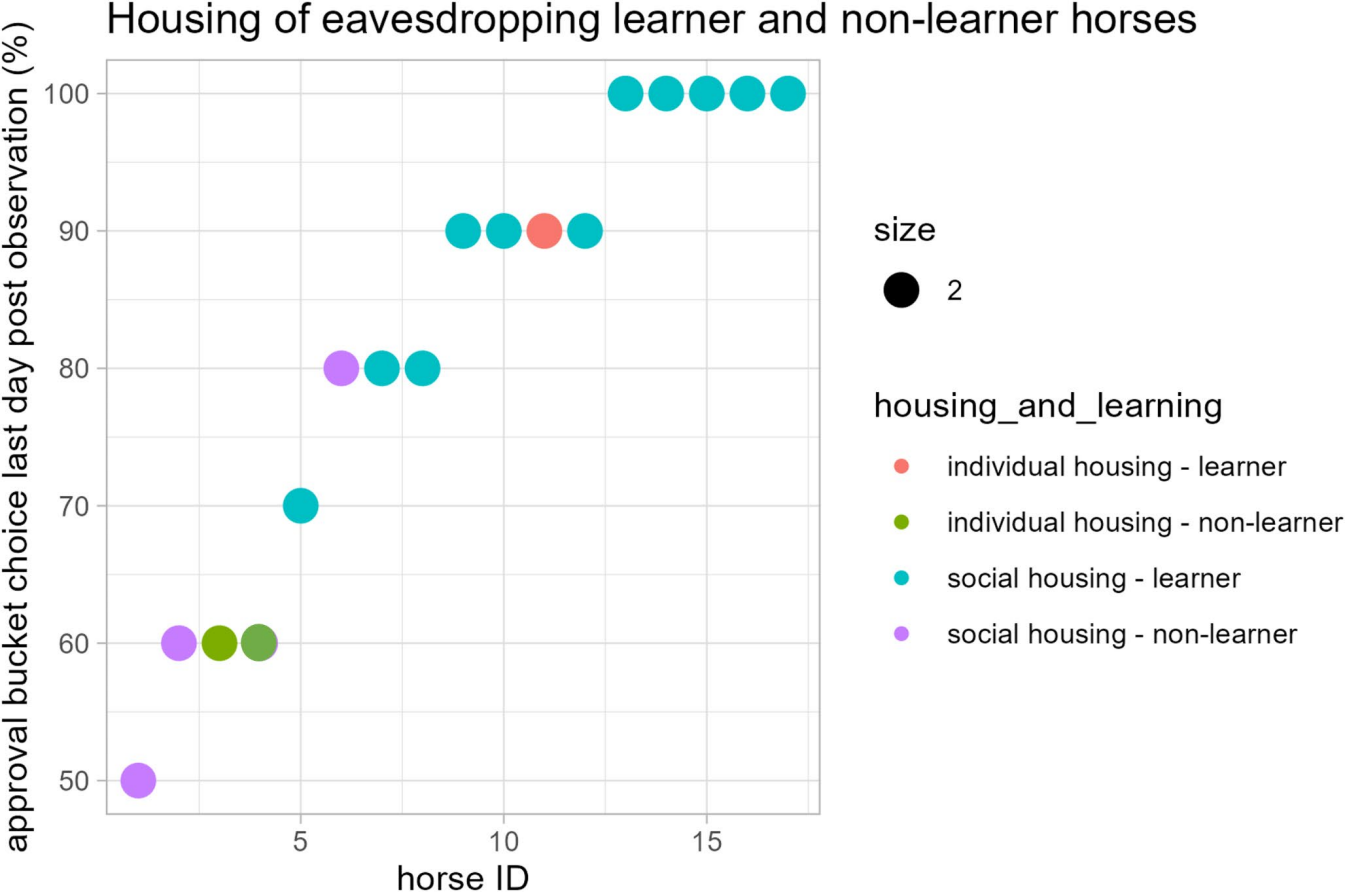
Housing conditions of horses learning to choose an approval bucket after observing human-human encounters. 12 of the 17 test horses significantly chose the approval bucket (red and blue; Regression analysis: all *p* < 0.05; Supplementary Information File S1). The test horses were kept either in social housing (blue and violet) or in individual housing (red and green). For an improved visualisation the dots are double sized

After observing the human-human feed bucket encounter 12 of the 17 horses learned to choose the individually assigned, approved feed bucket (Regression analysis, all *p* < 0.05, Figs. 1, 2 and 3; Table 1; Supplementary Information Tables S1, Supplementary Information File S1). Only 5 did not significantly learn to choose the approval bucket (Regression analysis, all *p* > 0.05, Supplementary Information File S1). One of the five horses reached performance criterion on day 7 and 8, but displayed an inconsistent performance all through the test (Regression analysis: *N* = 9, t = 1.97, *p* = 0.11), and was therefore considered a non-learner.

**Table 1:** Horse data and horse learning performance: **Horses’ data**: each horses’ sex, breed, age, housing condition, social rank and handling persons; **Pre-observation**: the horses’ % of choice for the approval bucket before observing a human-human encounter; **Post-observation**: the horses’ % of choice for the approval bucket after observing the human-human encounters

Horse Data							Pre-observation		Post-observation				
Horse	Sex horse	breed	Age horse	housing	rank ADI	Hand ling person E1 and stable	Approval bucket choice pre-observation (%)	Approval bucket choice 1st day post-observat ion (%)	Approval bucket choice 3rd day post-observat ion (%)	Approval bucket choice last day post observation (%)	Number of trials post-observation	Days to 2 × 80% criterion	Criterion 2 × 80% choice approval bucket
Beauty	female	Warmblood	4	social		5	40	50	80	50	76		0
Paula	female	Pony	13	individual		4	44	50	0	60	55		0
Goldi	female	Pony	20	individual		5	44	60	50	60	70		0
Rivaldo	male	Warmblood	15	social	0.754	1	50	57	25	60	54		0
Hektor	male	Pony	19	social	0.82	2	47	60	60	80	35	7	1
Gidion*	male	Pony	6	social	0.685	1	39	50	57	70	54		0
Wings*	male	Pony	11	social	0.453	2	33	20	20	80	35		0
Wesley*	male	Pony	21	social	0	3	17	0	70	80	42	5	1
Haylie*	female	Warmblood	4	social	0.436	1	50	0	40	90	41	7	1
Tin Elen*	female	Thouroughbred	15	social	1	3	44	60	90	90	40	4	1
La Luna*	female	Thouroughbred	24	individual		5	39	40	25	90	63		0
Winnie*	male	Pony	9	social	0.125	1	33	50	57	90	54	7	1
Tulipan*	male	Warmblood	10	social	0.642	2	0	0	80	100	35	6	1
Legenda*	female	Thouroughbred	10	social	0.587	2	13	0	60	100	35	7	1
Elly*	female	Pony	12	social	0.417	2	40	20	40	100	35	5	1
Askur*	male	Pony	12	social	0.225	2	47	60	80	100	35	3	1
Alrun*	male	Pony	15	social	0.313	2	0	20	80	100	35	4	1

* = learner (regression analysis for consistency in bucket choice: *p* < 0.05)

The likelihood of individual horses choosing the approved bucket and location (GEE: *N* = 794, Z = 6.605, *p* < 0.001) and the median group preference for the approved bucket and location (Friedman rank sum test: *N* = 17, X²_3_ = 29.58, *p* < 0.001; Fig. 2) increased over the test days (GEE: *N* = 794, Z = 6.08, *p* < 0.001). As a group, the horses’ performance was significantly higher on the last day than on the previous test days: pre-observation (Wilcoxon signed rank test: *N* = 17, V = 0, *p* < 0.001), 1st day post-observation (Wilcoxon signed rank test: *N* = 17, V = 0, *p* < 0.001), and 3rd day post-observation (Wilcoxon signed rank test: *N* = 17, V = 8, *p* = 0.002). However, the group’s performance did not differ significantly between the pre-observation and the post-observation days 1 and 3 (Wilcoxon signed rank test: *N* = 17, not significant after Bonferroni correction).

The social rank of the horses (ADI), their age, their sex, the number of trials they performed, and the strength of their bucket preference in the pre observation phase did not affect their bucket choice (Table 1; GEE: *N* = 794, all *p* > 0.05; Supplementary Information File S1). However, experimenter 1 (E1), who displayed the approval and disapproval, tended to affect the horses bucket choice (GEE: *N* = 794, Z = 1.86, *p* = 0.06). Furthermore, throughout the test, when considering all trials and all test days, the 14 horses kept in social housing chose the approval bucket in a higher percentage of trials on their test days, than the 3 horses kept in individual stabling (GEE: *N* = 794, Z = − 2.72, *p* = 0.007; Fig. 3; Table 1).

## Discussion

12 of 17 horses appeared to learn through eavesdropping on human-human disapproval and approval encounters regarding feeding at a specific feeding location. Even in the absence of those persons, they showed a significant change in their feeding preference in favour of the “approval” bucket and location and, therefore, meet definitions of social learning (Byrne 1994; Heyes 1994; Whiten et al. 2004). It seems the horses associated the human behaviour with the locations, not just with the people involved. This implies that social learning about the quality of feeding sites (Galef 1989) might set in when third parties eavesdrop on interactions of others. This has been proposed before (Nakamichi et al. 1998; Fiore et al. 2020), but has not been investigated in previous eavesdropping studies (Kundey et al. 2010; Freidin et al. 2013; Carballo et al. 2015; Jim et al. 2020). Such use of interspecies information could have a clear benefit for prey species, such as equids (Rendell et al. 2011) when learning about safe feeding sides, and when avoiding both predators and locations where predators might be present, but not visible (Ridley et al. 2013). Horses have previously been shown to return to preferred feeding locations, but to adjust their choice according to the group hierarchy (Krueger and Flauger 2008). We suggest that horses may also derive information pertaining to social feeding decisions from observing human interactions.

To learn from eavesdropping on the human-human encounter the horses could have used several learning mechanisms (Zajonc 1965; Heyes 1994; Whiten et al. 2004; Laland 2004). As suggested for dogs (Mersmann et al. 2011; Freidin et al. 2013; Jim et al. 2020) and horses in a previous study (Bernauer et al. 2020), we propose that learning was initiated by social enhancement (Heyes 1994; Whiten et al. 2004), was caused by persons interacting (i.e. stimulus enhancement) at a specific place (i.e. local enhancement), and may have shaped the horses’ preferences for a feeding location, as shown in rats (Galef 1989). The local enhancement may have initiated individual trial and error learning, as discussed for dogs (Mersmann et al. 2011) and pigs (Veit et al. 2022). Furthermore, repeatedly observing the interaction at the feeding buckets may have initiated social conditioning for the approved feeding location (Whiten et al. 2004). As persons who were well known to the horses displayed the approval and disapproval signals, previous conditioning to the particular demonstrator’s approval and disapproval signals may have combined with the social conditioning for the approval buckets’ location (Veit et al. 2022). However, as the horses showed a lasting change in feeding bucket and feeding location preference after eavesdropping in the absence of the interacting persons, i.e. not towards the interacting persons themselves, the horses may even have understood the meaning of the persons’ signals regarding the value of the feeding locations. They may have imitated the feeding location choice (Whiten and Ham 1992; Zentall 2006; Byrne 2009), by choosing the same place observed in the approval demonstration. Social learning may have resulted in the change of feeding preferences being expressed even in the absence of the demonstrating persons (Whiten et al. 2004), as observed in potato washing Japanese macaques (Nakamichi et al. 1998; Fiore et al. 2020).

The management system under which the horses were living was an important factor. The horses kept in social housing systems, with greater social experience with conspecifics than horses kept in individual housing, appeared to infer in a larger percentage of their performance trials that the information spread unintentionally by the human-human interaction might also apply to them. This provides further support for the intraspecies and interspecies ‘social intelligence hypothesis’ (Byrne and Whiten 1988; Speechley et al. 2024), which suggests that social experience within the species is a crucial factor for the development of cognitive skills such as using social cues (Liehrmann et al. 2023) and social learning within and across species (Krueger and Heinze 2008). In this study, the less socially experienced horses, i.e. those kept in individual housing, adapted their behaviour in a smaller percentage of trials after eavesdropping on the human-human interaction (Krueger and Heinze 2008; Carballo et al. 2015), and this could also have important implications for animal welfare and training (Dorey et al. 2014). Social contact and companionship with conspecifics are established basic needs for horses’ welfare (Krueger et al. 2021). A change from social to individual housing (Marr et al. 2020) was shown to increase stress in young horses, even to the degree of a slight immune depression (Schmucker et al. 2022). We therefore support the finding of Liehrmann et al. (2023) that insufficient social contact could compromise the development of ‘social intelligence’ (Byrne and Whiten 1988; Speechley et al. 2024), and therefore the development of intra- and interspecific social learning skills in horses (Krueger and Heinze 2008; Krueger et al. 2014; Schuetz et al. 2017; Bernauer et al. 2020), with socially kept horses learning more readily from observation.

Previously, lower ranking, younger horses have been shown to be more exploratory than older horses (Krueger et al. 2014), and to be more easily influenced by human signalling (Krueger and Heinze 2008; Schuetz et al. 2017; Bernauer et al. 2020). While age and social rank were not significant factors in this study, the signalling of the familiar experimenter did have an impact on the horses’ performance. Future research should investigate these, and other variables that may affect the animals’ choices in more detail, including the familiarity of the human demonstrators (Carballo et al. 2015; Bernauer et al. 2020), the animals’ social experience with both humans and conspecifics (Carballo et al. 2015; Bernauer et al. 2020), the signals provided by the demonstrating persons (Miklósi and Soproni 2006; Maros et al. 2008; Proops and McComb 2010; Proops et al. 2013), the type of training (Dorey et al. 2014), and personality traits, such as pessimism and optimism (Löckener et al. 2016; Marr et al. 2018). Some horses may be more consistent in making choices, as has been shown in optimistic rather than in pessimistic horses (Marr et al. 2018), and some more flexible, as has been observed in pigs (Asher et al. 2016). When horses were moved between individual and social housing, they tended to make pessimistic choices when kept in individual housing and more optimistic choices when kept in social housing (Löckener et al. 2016). Follow up studies should include detailed personality assessments.

Future studies should investigate in more detail whether the horses respond to the interaction between two people or to the signals provided by the person demonstrating the approval and disapproval. A ghost control situation, with the demonstrator displaying an approval and disapproval but without any responding person, would be appropriate (Marshall-Pescini 2011). If horses would mostly respond to the signals of the demonstrator, they would also change their bucket choice after observing the ghost control trials, without observing any responding person. Furthermore, future research should investigate the durability of this preference change in the absence of repeated human demonstrations to see whether long-term, interspecies social learning (Whiten et al. 2004), sets in after third party eavesdropping on human-human interactions (Freidin et al. 2013). Longer-term behaviour changes would have important implications for the unintentional impact of human interactions on interspecies communication (Healy and Jones 2002; Laland and O’Brien 2011; Lee and Thornton 2021; Sueur and Huffman 2024).

## Conclusion

The present study can be considered a pilot experiment in a controlled setting, which indicates that animals may change feeding strategies after eavesdropping on human-human demonstrations, even in the absence of the demonstrators. It is particularly interesting that the horses that were in single housing, and therefore not in a social environment with conspecifics, adapted to the human-human demonstration less consistently. Social contact is an established basic need in horses (Krueger et al. 2021) and if this need is compromised, it appears to have an effect on the horse’s ability to learn and adapt (Liehrmann et al. 2023). This has major implications for welfare and training for domestic horses (Marr et al. 2020; Schmucker et al. 2022).

More generally, as a variety of domesticated animals (Asher et al. 2016) (pigs and dogs: Gerencsér et al. 2019; horses: Maros et al. 2008; Liehrmann et al. 2023) and wild animals (Pampas foxes, *Lycalopex gymnocercus*, Barrera et al. 2012) use human pointing gestures to find food in direct interactions between animals and humans, the unintentional impact of humans on creating interspecies communication traditions, and especially on unintentional cultural co-evolution between humans and domestic as well as wild animals (Laland and O’Brien 2011; Lee and Thornton 2021; Spottiswoode and Wood 2023; Sueur and Huffman 2024) needs to be reconsidered.

## Electronic supplementary material

Below is the link to the electronic supplementary material.

Supplementary Material 1

Supplementary Material 2

## Data Availability

All data is provided within the manuscript or supplementary information files.

## Declarations

### Declaration of AI use

We have not used AI-assisted technologies in creating this article.
