# Supplementary material for: Learning from eavesdropping on human-human encounters changes feeding location choice in horses (Equus Caballus)
Source: Anim Cogn. 2025 Mar 17;28(1):23. doi: 10.1007/s10071-025-01946-1 (PMC11913996; doi:10.1007/s10071-025-01946-1)
Supplement: Supplementary file 1 — Supplementary Material 1 [file 10071_2025_1946_MOESM1_ESM.pdf]

Supplementary Information

Learning from eavesdropping on human-human encounters changes feeding location choice in horses (*Equus Caballus*)

Konstanze Krueger, Anika Roll, Anna J. Beyer, Angela Föll, Maren Bernau, Kate Farmer

File S1.

Data analysis. Statistical analysis and the depiction of the data (table 1; Supplementary Information Tables S1) was conducted using R Studio (version 0.99.484, Boston MA, USA) of the R-Project statistical environment (R Development Core Team, version 4.4.2) and the package lme4. Most of the data were not normally distributed (K-S test). A Kendall's rank correlation tau test was applied for an inter-observer reliability assessment between the experimenters and a naïve person. Regression analyses were used to evaluate the horses' performances at individual level. A Friedman rank sum test and Wilcoxon signed rank tests were used to compare the horses' performances at group level. We applied a nested GEE for multivariate factor analysis. The GGE was nested for the individual horses as a random effect, all other factors under consideration were not truly independent from the experimental design and were therefore included as fixed factors. The model with the best fit (the model with the lowest information loss versus the lowest clustering, quoted with the lowest Akaike information criterion, AIC) was chosen after stepwise removal of factors. A Generalised Estimation Equation (GEE) to analyse the likelihood of each factor affecting the choice for a certain feed bucket throughout the total trials (N = 794) was applied, with the following formular: GEE (formula = choice.disapproval.vs.approval bucket ~ (ADI.horse + age.horse + day + handling.person.E1 + housing + sex.horse + strength.bucket.preference.habituation + trail) %in% horse.num, family = binomial(logit), data = Dataset). For the full and reduced model see Supplementary Information File S1. All tests were two sided, and the significance level was set at $p \leq 0.05$. After multiple testing, the significance level was adjusted with a Sequential Bonferroni Correction after Holm (1979) and only p-values that were below the corrected significance level were considered to be significant.

1. Inter-observer reliability assessment

Kendall's rank correlation tau

data: choice.disapproved.vs.approved...18 and choice.disapproved.vs.approved...19
z = 28.16, p-value < 2.2e-16
alternative hypothesis: true tau is not equal to 0
sample estimates:
tau 1

2. Regression analyses for evaluating the horses' performances at individual level

```
Call:
lm(formula = Gidion_approval_yellow_percent ~ Tag, data = Dataset)

Residuals:
   Min     1Q Median     3Q    Max 
-5.095 -3.080 -1.702  1.971  9.730 

Coefficients:
            Estimate Std. Error t value Pr(>|t|)    
(Intercept)   36.498      4.085   8.934 0.000110 ***
Tag            4.825      0.809   5.964 0.000996 ***
---
Signif. codes:  0 '***' 0.001 '**' 0.01 '*' 0.05 '.' 0.1 ' ' 1

Residual standard error: 5.243 on 6 degrees of freedom
  (1 Beobachtung als fehlend gelöscht)
Multiple R-squared:  0.8556,	Adjusted R-squared:  0.8316 
F-statistic: 35.57 on 1 and 6 DF,  p-value: 0.0009957


Call:
lm(formula = Haylie_approval_yellow_percent ~ Tag, data = Dataset)

Residuals:
```

```
   Min     1Q Median     3Q    Max 
-28.93 -11.28  -3.69  11.58  40.83 

Coefficients:
            Estimate Std. Error t value Pr(>|t|)  
(Intercept)  -0.7143    18.2374  -0.039   0.9700  
Tag           9.8810     3.6115   2.736   0.0339 *
---
Signif. codes:  0 '***' 0.001 '**' 0.01 '*' 0.05 '.' 0.1 ' ' 1

Residual standard error: 23.41 on 6 degrees of freedom
  (1 Beobachtung als fehlend gelöscht)
Multiple R-squared:  0.5551,	Adjusted R-squared:  0.4809 
F-statistic: 7.485 on 1 and 6 DF,  p-value: 0.03392


Call:
lm(formula = Rivaldo_approval_yellow_percent ~ Tag, data = Dataset)

Residuals:
    Min      1Q  Median      3Q     Max 
-23.321  -9.297   3.144   9.308   9.796 

Coefficients:
            Estimate Std. Error t value Pr(>|t|)   
(Intercept)  46.3672     9.1065   5.092  0.00141 **
Tag           0.4883     1.6183   0.302  0.77160   
---
Signif. codes:  0 '***' 0.001 '**' 0.01 '*' 0.05 '.' 0.1 ' ' 1

Residual standard error: 12.53 on 7 degrees of freedom
Multiple R-squared:  0.01284,	Adjusted R-squared:  -0.1282 
F-statistic: 0.09106 on 1 and 7 DF,  p-value: 0.7716


Call:
lm(formula = Winnie_approval_yellow_percent ~ Tag, data = Dataset)

Residuals:
     Min       1Q   Median       3Q      Max 
-12.2314  -3.3375  -0.8488   1.7402  17.1235 

Coefficients:
            Estimate Std. Error t value Pr(>|t|)   
(Intercept)   25.861      7.772   3.327  0.01586 * 
Tag            8.145      1.539   5.292  0.00184 **
---
Signif. codes:  0 '***' 0.001 '**' 0.01 '*' 0.05 '.' 0.1 ' ' 1

Residual standard error: 9.974 on 6 degrees of freedom
  (1 Beobachtung als fehlend gelöscht)
Multiple R-squared:  0.8236,	Adjusted R-squared:  0.7942 
F-statistic: 28.01 on 1 and 6 DF,  p-value: 0.001844


Call:
lm(formula = Alrun_approval_yellow_percent ~ day, data = Dataset)

Residuals:
      1       2       3       4       5       6       7 
-20.000   8.571  17.143   5.714  -5.714   2.857  -8.571 

Coefficients:
            Estimate Std. Error t value Pr(>|t|)   
(Intercept)   28.571     11.429   2.500  0.05449 . 
day           11.429      2.556   4.472  0.00657 **
---
Signif. codes:  0 '***' 0.001 '**' 0.01 '*' 0.05 '.' 0.1 ' ' 1

Residual standard error: 13.52 on 5 degrees of freedom
  (1 Beobachtung als fehlend gelöscht)
Multiple R-squared:    0.8,	Adjusted R-squared:   0.76 
F-statistic:    20 on 1 and 5 DF,  p-value: 0.006566


Call:
lm(formula = day ~ Askur_approval_blue_percent, data = Dataset)
```

```
Residuals:
   1    2    3    4    5    6    7 
 0.2 -1.6 -0.6  0.4  1.4 -0.4  0.6 

Coefficients:
                            Estimate Std. Error t value Pr(>|t|)  
(Intercept)                  -7.6000     2.6245  -2.896  0.03396 * 
Askur_approval_blue_percent   0.1400     0.0313   4.472  0.00657 **
---
Signif. codes:  0 '***' 0.001 '**' 0.01 '*' 0.05 '.' 0.1 ' ' 1

Residual standard error: 1.058 on 5 degrees of freedom
  (2 Beobachtungen als fehlend gelöscht)
Multiple R-squared:    0.8,	Adjusted R-squared:   0.76 
F-statistic:    20 on 1 and 5 DF,  p-value: 0.006566


Call:
lm(formula = day ~ Elly_approval_blue_percent, data = Dataset)

Residuals:
      1       2       3       4       5       6       7 
-0.1667 -0.5833 -1.0000  1.4167 -0.4167  0.5833  0.1667 

Coefficients:
                           Estimate Std. Error t value Pr(>|t|)   
(Intercept)                -0.25000    0.83630  -0.299  0.77702   
Elly_approval_blue_percent  0.07083    0.01277   5.545  0.00262 **
---
Signif. codes:  0 '***' 0.001 '**' 0.01 '*' 0.05 '.' 0.1 ' ' 1

Residual standard error: 0.8851 on 5 degrees of freedom
  (2 Beobachtungen als fehlend gelöscht)
Multiple R-squared:  0.8601,	Adjusted R-squared:  0.8321 
F-statistic: 30.74 on 1 and 5 DF,  p-value: 0.00262


Call:
lm(formula = Hektor_approval_yellow_percent ~ day, data = Dataset)

Residuals:
  1   2   3   4   5   6   7 
 15 -10   5 -20  -5  10   5 

Coefficients:
            Estimate Std. Error t value Pr(>|t|)  
(Intercept)   40.000     11.339   3.528   0.0168 *
day            5.000      2.535   1.972   0.1056  
---
Signif. codes:  0 '***' 0.001 '**' 0.01 '*' 0.05 '.' 0.1 ' ' 1

Residual standard error: 13.42 on 5 degrees of freedom
  (1 Beobachtung als fehlend gelöscht)
Multiple R-squared:  0.4375,	Adjusted R-squared:  0.325 
F-statistic: 3.889 on 1 and 5 DF,  p-value: 0.1056


Call:
lm(formula = Legenda_approval_blue_percent ~ day, data = Dataset)

Residuals:
      1       2       3       4       5       6       7 
-16.429  10.000  16.429   2.857 -10.714  -4.286   2.143 

Coefficients:
            Estimate Std. Error t value Pr(>|t|)   
(Intercept)    2.857     10.595   0.270  0.79818   
day           13.571      2.369   5.729  0.00227 **
---
Signif. codes:  0 '***' 0.001 '**' 0.01 '*' 0.05 '.' 0.1 ' ' 1

Residual standard error: 12.54 on 5 degrees of freedom
  (1 Beobachtung als fehlend gelöscht)
Multiple R-squared:  0.8678,	Adjusted R-squared:  0.8413 
F-statistic: 32.82 on 1 and 5 DF,  p-value: 0.002269


Call:
```

```
lm(formula = Tulipan_approval_blue_percent ~ day, data = Dataset)

Residuals:
      1       2       3       4       5       6       7 
-30.000  17.143  24.286  -8.571  18.571 -14.286  -7.143 

Coefficients:
            Estimate Std. Error t value Pr(>|t|)  
(Intercept)   17.143     18.736   0.915   0.4022  
day           12.857      4.189   3.069   0.0278 *
---
Signif. codes:  0 '***' 0.001 '**' 0.01 '*' 0.05 '.' 0.1 ' ' 1

Residual standard error: 22.17 on 5 degrees of freedom
  (1 Beobachtung als fehlend gelöscht)
Multiple R-squared:  0.6532,	Adjusted R-squared:  0.5839 
F-statistic: 9.419 on 1 and 5 DF,  p-value: 0.02782


Call:
lm(formula = Wings_approval_blue_percent ~ day, data = Dataset)

Residuals:
      1       2       3       4       5       6       7 
 12.857   2.857  -7.143 -17.143  -7.143   2.857  12.857 

Coefficients:
            Estimate Std. Error t value Pr(>|t|)   
(Intercept)   -2.857     10.302  -0.277  0.79261   
day           10.000      2.304   4.341  0.00742 **
---
Signif. codes:  0 '***' 0.001 '**' 0.01 '*' 0.05 '.' 0.1 ' ' 1

Residual standard error: 12.19 on 5 degrees of freedom
  (1 Beobachtung als fehlend gelöscht)
Multiple R-squared:  0.7903,	Adjusted R-squared:  0.7484 
F-statistic: 18.85 on 1 and 5 DF,  p-value: 0.00742



Call:
lm(formula = Beauty.approval._yellow_percent ~ Tag, data = Dataset)

Residuals:
    Min      1Q  Median      3Q     Max 
-17.956  -6.556  -6.256  13.244  23.344 

Coefficients:
            Estimate Std. Error t value Pr(>|t|)    
(Intercept)   57.056     10.397   5.488 0.000918 ***
Tag           -0.100      1.848  -0.054 0.958347    
---
Signif. codes:  0 '***' 0.001 '**' 0.01 '*' 0.05 '.' 0.1 ' ' 1

Residual standard error: 14.31 on 7 degrees of freedom
Multiple R-squared:  0.0004183,	Adjusted R-squared:  -0.1424 
F-statistic: 0.00293 on 1 and 7 DF,  p-value: 0.9583


Call:
lm(formula = Goldi_approval_yellow_percent ~ Tag, data = Dataset)

Residuals:
    Min      1Q  Median      3Q     Max 
-37.976  -3.623   0.119  13.248  18.895 

Coefficients:
            Estimate Std. Error t value Pr(>|t|)   
(Intercept)   66.140     14.376   4.601  0.00248 **
Tag           -3.129      2.555  -1.225  0.26022   
---
Signif. codes:  0 '***' 0.001 '**' 0.01 '*' 0.05 '.' 0.1 ' ' 1

Residual standard error: 19.79 on 7 degrees of freedom
Multiple R-squared:  0.1765,	Adjusted R-squared:  0.05887 
F-statistic:   1.5 on 1 and 7 DF,  p-value: 0.2602
```

```
Call:
lm(formula = La.Luna_approval_blue_percent ~ Tag, data = Dataset)

Residuals:
    Min      1Q  Median      3Q     Max 
-21.219  -4.182   2.809   7.762  13.643 

Coefficients:
            Estimate Std. Error t value Pr(>|t|)   
(Intercept)   18.257      8.891   2.053  0.07914 . 
Tag            6.991      1.580   4.424  0.00306 **
---
Signif. codes:  0 '***' 0.001 '**' 0.01 '*' 0.05 '.' 0.1 ' ' 1

Residual standard error: 12.24 on 7 degrees of freedom
Multiple R-squared:  0.7366,	Adjusted R-squared:  0.699 
F-statistic: 19.58 on 1 and 7 DF,  p-value: 0.003065


Call:
lm(formula = Paula_approval_blue_percent ~ Tag, data = Dataset)

Residuals:
    Min      1Q  Median      3Q     Max 
-38.512  -6.124   1.339  13.540  16.563 

Coefficients:
            Estimate Std. Error t value Pr(>|t|)  
(Intercept)   28.362     13.587   2.087   0.0753 .
Tag            2.537      2.415   1.051   0.3282  
---
Signif. codes:  0 '***' 0.001 '**' 0.01 '*' 0.05 '.' 0.1 ' ' 1

Residual standard error: 18.7 on 7 degrees of freedom
Multiple R-squared:  0.1363,	Adjusted R-squared:  0.01287 
F-statistic: 1.104 on 1 and 7 DF,  p-value: 0.3282


Call:
lm(formula = Tin.Ellen_approval_yellow_percent ~ Tag, data = Dataset)

Residuals:
     1      2      3      4      5 
-0.224  3.224 -8.888  9.000 -3.112 

Coefficients:
            Estimate Std. Error t value Pr(>|t|)  
(Intercept)   32.552      8.127   4.005   0.0279 *
Tag           12.112      2.450   4.943   0.0159 *
---
Signif. codes:  0 '***' 0.001 '**' 0.01 '*' 0.05 '.' 0.1 ' ' 1

Residual standard error: 7.749 on 3 degrees of freedom
  (4 Beobachtungen als fehlend gelöscht)
Multiple R-squared:  0.8906,	Adjusted R-squared:  0.8542 
F-statistic: 24.43 on 1 and 3 DF,  p-value: 0.01588


Call:
lm(formula = Wesley_approval_blue_percent ~ Tag, data = Dataset)

Residuals:
      1       2       3       4       5       6 
  6.033 -26.827  16.983  10.793   4.603 -11.587 

Coefficients:
            Estimate Std. Error t value Pr(>|t|)  
(Intercept)   -5.553     16.889  -0.329   0.7588  
Tag           16.190      4.337   3.733   0.0202 *
---
Signif. codes:  0 '***' 0.001 '**' 0.01 '*' 0.05 '.' 0.1 ' ' 1

Residual standard error: 18.14 on 4 degrees of freedom
  (3 Beobachtungen als fehlend gelöscht)
Multiple R-squared:  0.777,	Adjusted R-squared:  0.7213 
F-statistic: 13.94 on 1 and 4 DF,  p-value: 0.02024
```

3. **A Friedman rank sum test and Wilcoxon signed rank tests to compare the horses' performances at group level.**

```
Medians:
approval.bucket..choice.pre.observation....
                                                 39.99
approval.bucket.choice.1st.day.post.observation....
                                                 50.00
approval.bucket.choice.3rd.day.post.observation....
                                                 57.00
approval.bucket.choice.last.day.post.observation....
                                                 90.00


Friedman rank sum test

data:  .Responses
Friedman chi-squared = 29.577, df = 3, p-value = 0.000001694


Wilcoxon signed rank test with continuity correction

data:  approval.bucket..choice.pre.observation.... and approval_bucket_choi
ce_last_day_post_observation
V = 0, p-value = 0.0003198 (significant after Bonferronie correction)

Wilcoxon signed rank test with continuity correction


data:  approval.bucket.choice.1st.day.post.observation.... and approval_buc
ket_choice_last_day_post_observation
V = 0, p-value = 0.000714 (significant after Bonferronie correction)

Wilcoxon signed rank test with continuity correction

data:  approval.bucket.choice.3rd.day.post.observation.... and approval_buc
ket_choice_last_day_post_observation
V = 8, p-value = 0.00204 (significant after Bonferronie correction)


Wilcoxon signed rank test with continuity correction

data:  approval.bucket..choice.pre.observation.... and approval.bucket.choi
ce.3rd.day.post.observation....
V = 29.5, p-value = 0.04935 (not significant after Bonferronie correction)


Wilcoxon signed rank test with continuity correction

data:  approval.bucket.choice.1st.day.post.observation.... and approval.buc
ket.choice.3rd.day.post.observation....
V = 27, p-value = 0.06469 (not significant)

Wilcoxon signed rank test with continuity correction

data:  approval.bucket..choice.pre.observation.... and approval.bucket.choi
ce.1st.day.post.observation....
V = 59, p-value = 0.6599 (not significant)
```

4. **Complete Multivariate Model**

```
Call:
glm(formula = choice.disapproved.vs.approved ~ (ADI.horse + age.horse +
    day + handling.person.E1 + housing + sex.horse + strength.bucket.preference.habituation +
    trail) %in% horse.num, family = binomial(logit), data = Dataset)

Coefficients:
                                                 Estimate Std. Error z value Pr(>|z|)
(Intercept)                                      0.257199   0.267145   0.963   0.3357
ADI.horse:horse.num                             -0.015168   0.057474  -0.264   0.7919
age.horse:horse.num                             -0.002067   0.002953  -0.700   0.4839
day:horse.num                                    0.054575   0.008255   6.611 3.82e-11 ***
handling.person.E1:horse.num                     0.041731   0.041147   1.014   0.3105
housing:horse.num                               -0.249591   0.115958  -2.152   0.0314 *
sex.horse:horse.num                              0.038468   0.039405   0.976   0.3290
strength.bucket.preference.habituation:horse.num 0.015232   0.034903   0.436   0.6625
trail:horse.num                                  0.004950   0.005811   0.852   0.3943
---
Signif. codes:  0 '***' 0.001 '**' 0.01 '*' 0.05 '.' 0.1 ' ' 1

(Dispersion parameter for binomial family taken to be 1)

    Null deviance: 695.12  on 529  degrees of freedom
Residual deviance: 637.21  on 521  degrees of freedom
  (264 Beobachtungen als fehlend gelöscht)
AIC: 655.21
```

Model with lowest AIC after reducing factors

```
Call:
glm(formula = choice.disapproved.vs.approved ~ (day + handling.person.E1 +
    housing + ADI.horse) %in% horse.num, family = binomial(logit),
    data = Dataset)

Coefficients:
                              Estimate Std. Error z value Pr(>|z|)
(Intercept)                   0.277345   0.244718   1.133  0.25708
day:horse.num                 0.054312   0.008223   6.605 3.98e-11 ***
handling.person.E1:horse.num  0.056612   0.030495   1.856  0.06339 .
housing:horse.num            -0.275956   0.101443  -2.720  0.00652 **
ADI.horse:horse.num           0.009632   0.036604   0.263  0.79245
---
Signif. codes:  0 '***' 0.001 '**' 0.01 '*' 0.05 '.' 0.1 ' ' 1

(Dispersion parameter for binomial family taken to be 1)

    Null deviance: 695.12  on 529  degrees of freedom
Residual deviance: 639.39  on 525  degrees of freedom
  (264 Beobachtungen als fehlend gelöscht)
AIC: 649.39

Number of Fisher Scoring iterations: 4
```
